# Preliminary Study on β3-Adrenoreceptor as Predictor Marker of Relapse in Ewing Sarcoma Patients

**DOI:** 10.3390/biomedicines8100413

**Published:** 2020-10-13

**Authors:** Maura Calvani, Marina Vignoli, Giovanni Beltrami, Amada Pasha, Perla Scalini, Sara Ciullini Mannurita, Stefania Cardellicchio, Luca Coccoli, Cecilia Cecchi, Emanuela De Marco, Laura Luti, Sayla Bernasconi, Luca Filippi, Gabriella Casazza, Angela Tamburini, Claudio Favre

**Affiliations:** 1 Division of Pediatric Oncology/Hematology, Meyer University Children’s Hospital, 50139 Florence, Italy; marina.vignoli@unifi.it (M.V.); amada.pasha@stud.unifi.it (A.P.); perla.scalini@meyer.it (P.S.); sara.ciullinimannurita@meyer.it (S.C.M.); stefania.cardellicchio@meyer.it (S.C.); cecilia.cecchi@meyer.it (C.C.); angela.tamburini@meyer.it (A.T.); claudio.favre@meyer.it (C.F.); 2 Department of Health Sciences, University of Florence, 50139 Florence, Italy; 3 Division of Pediatric Oncological Orthopedics, Meyer University Children’s Hospital, 50139 Florence, Italy; giovanni.beltrami@meyer.it; 4 Paediatric Hematology Oncology, Bone Marrow Transplant, S. Chiara University Hospital of Pisa, 56126 Pisa, Italy; l.coccoli@ao-pisa.toscana.it (L.C.); e.demarco@ao-pisa.toscana.it (E.D.M.); l.luti@ao-pisa.toscana.it (L.L.); s.bernasconi@ao-pisa.toscana.it (S.B.); g.casazza@ao-pisa.toscana.it (G.C.); 5 Neonatal Intensive Care Unit, Medical Surgical Fetal-Neonatal Department, Meyer University Children’s Hospital, 50139 Florence, Italy; luca.filippi@meyer.it

**Keywords:** Ewing sarcoma, β3-adrenergic receptors, circulating tumour cells

## Abstract

Ewing sarcoma (EWS) is a paediatric aggressive malignant tumour of bones and soft tissues. Multidisciplinary chemotherapies, surgical resection, and radiation represent the only strategies counteracting the disease, however spreading and relapse of disease still remain a clinical issue. Circulating tumour cells (CTCs) are an important feature of EWS but the prognostic significance has not been, yet, clarified. CTCs have been found both in patients with localized disease and in those who recur or metastasize. The identification of markers that can detect recurrences and metastasis remains an important challenge for research. Unfortunately, even most of patients with localized cancer relapsed and the reason has not yet been fully understood. In this clinical study on EWS patients, we evaluated the expression of CD99 antigen and beta-3 adrenergic receptor (β3-AR) on CTCs and bioptic derived cells by flow cytometry. The preliminary data revealed a higher β3-AR expression on cells derived from metastatic or relapsed patients, suggesting a role for the β3-AR as a possible predictive maker of disease recurrence in both patients with metastatic and localized disease.

## 1. Introduction

Ewing sarcoma (EWS) is a highly aggressive and metabolically active malignant tumour of soft tissue and bones, that primarily occurs in adolescents and young adults [1,2]. EWS is a member of Ewing family tumours (ESFT) which include peripheral primitive neuroectodermal tumours (PNET) and Askin tumours [3]. EWS is the second most common paediatric malignant bone tumour that develops not only in osseous sites but also in extra-skeletal soft tissues and represents 2% of all childhood cancers [4,5]. According to literature, the estimated five-year survival in cases of localized disease, i.e., non-metastatic cases, is between 50% and 70% [6,7]. Survival rates have increased, approximately, up to 70% through implementation of intensive multi-drug systemic chemotherapy regimen, along with surgery and/or radiotherapy [8]. However, even after undergoing intensive multi-modality treatment (that include also allogeneic haematopoietic stem cell transplantation) the prognosis for patients with metastatic or recurrent disease remains unfavourable [5,9]. Current researches have focused on identifying and targeting specific pathways and genes that would have a crucial role in the development of therapeutic resistance.

In 85% of cases EWS is associated with, the t(11;22)(q24:q12) chromosomal translocation that generates the fusion of *EWS* gene-5′ segment with the 3′ segment of the ETS family gene *FLI-1* [10]. The EWS-FLI1 fusion protein is an oncogenic chimeric transcription factor which plays a key role in EWS pathogenesis, and that can cause neoplastic transformation by deregulating the expression of hundreds of genes [11]. The EWS-FLI1 fusion protein has been found only in tumour cells. Due to this specificity, therapy targeting the EWS-FLI1 protein should avoid non-specific toxicities. An attractive tumour target expressed on the surface of EWS cells is represented by CD99 antigen, which is a heavily O-glycosylated transmembrane protein with a molecular weight of 32 kDa that results to be expressed in Ewing sarcoma [12], synovial sarcoma (SS), and low-grade fibromyxoid sarcoma (LGFMS) [13]; the down-regulation of CD99 in EWS cell lines reduced their ability to form tumours and bone metastases in immunodeficient mice and decreased their tumourigenic and metastatic features in vitro [13]. CD99 antigen can be a useful marker for the detection and isolation of circulating tumour cells (CTCs) in EWS [14]. CTCs were first described in the peripheral blood (PB) of cancer patients in 1955 [15]. They have been identified mainly in PB of epithelial tumours patients because of the surface expression of epithelial cell adhesion molecule (EpCAM), a marker for CTC enrichment and isolation. Despite CTCs having been found in PB of EWS patients, their presence has not been associated with metastasis and outcome, since, until now, there are not evidences that tumour cells detected in patients PB have the ability to give rise to metastasis [16]. Multiple studies confirmed that CTCs identified in PB do not have a clear association with metastasis and outcome [17,18,19].

Recently, since EWS cells are susceptible to an increased oxidative stress, beta-3-adrenergic receptor (β3-AR) has been proposed as a putative target for a possible strategy in cancer therapy due to its ability to induce apoptosis in EWS cell lines by increasing the level of reactive oxygen species (ROS) [20]. Most of conventional drugs used in EWS affect ROS balance by increasing the level of ROS over the toxic threshold to kill cancer cells. Among β-ARs, the β3-AR is the last identified member of this receptor family. It was shown to regulate lipolysis and thermogenesis [21]. β3-ARs became incredibly attractive in cancer biology because of their role in reducing tumour growth and metastasis. β3-ARs are highly expressed in embryonic tissue [22], but in adult healthy human tissues exhibit a restricted expression (myocardium, retina, myometrium, adipose tissue, gallbladder, brain, urinary bladder, and blood vessels). In infantile haemangioma (a benign tumour of childhood) and in a range of malignant tumours (such as melanoma, astrocytoma, colon cancer, breast cancer, and leukaemia) considerable amounts of β3-ARs at the mRNA and/or protein level have been observed [23]. Cancer growth, recruitment of circulating stromal cell precursors to the tumour sites, and enhancement of stem cell traits have been associated with overexpression of β3-ARs [24]. Recent literature reported a role of β3-ARs on cancer progression and dissemination [25]. Over the years, the relationship between β-ARs and cancer initiation and progression has been well established, including: inflammation, angiogenesis, cell motility and trafficking, apoptosis, and cellular immune response. Many studies showed the overexpression of β-ARs across multiple cancer types and the pharmacological inhibition of the β-ARs with beta blockers as anticancer agents supported the evidence that β-blockers contribute to improved survival and decrease tumour proliferation and progression in multiple cancer types [26,27,28]. Dal Monte et al. showed the involvement of β3-ARs in melanoma initiation and progression and the potential role of β3-ARs blockade to contrast tumour cells proliferation [25]. They have reported that the treatment with the specific β3-ARs antagonist SR59230A is effective in reducing melanoma angiogenesis and growth and in inducing apoptosis. In addition, interesting results were observed about the effect of β-adrenoreceptor blockade on the number and activity of immune cell sub-populations (Treg, NK, CD8, MDSC, macrophages, and neutrophils). The β-adrenoreceptor blockade, both with antagonists (propranolol and SR59230A) and specific siRNAs, suggested the β3-ARs involvement in immune-tolerance [29]. Moreover, β3-ARs blockade can induce a switch from stemness features to a neuronal differentiation phenotype leading to a strong tumour growth reduction in neuroblastoma cells [30]. For future therapeutic modality, it is necessary to target not just the localized tumour cells, but also, residual tumour cells in the body, either in the form of circulating tumour cells or cancer stem-like cells. Evidences have suggested that the persistence of cancer stem cells can partially account for radio-resistance, chemo-resistance, and overall tumour invasion and recurrence in many cancers including sarcomas [31,32,33]. Since Calvani et al. have demonstrated the association between β3-ARs and the recruitment of circulating stromal cell precursors to the tumour sites and enhancement of stem cell traits [24], β3-ARs could be an interesting target for cancer therapy.

However, additional studies on β3-ARs blockers are necessary since currently the most widely used β3-ARs antagonists are L-748,337 and SR59230A, but they have some limitations. SR59230A was previously considered as a selective β3-adrenoceptor antagonist, but a similar affinity was demonstrated for all the three subtypes [34,35]. Another issue is that SR59230A can act as a partial agonist, and the degree of partial agonism depends on the model system. Moreover, in some systems, SR59230A acts as a full agonist [36].

We are conducting a clinical study on biological samples derived from EWS patients, evaluating the expression of some markers and their association with the disease progression and outcome. Here we report the preliminary results of the analysis of PB cells obtained from 20 EWS patients between 2018 and 2020. The study focused on the evaluation of β3-AR expression on total cells and on CD45^−^CD99^+^ cancer cells present in PB and on the analysis of the association with the clinical status of the patient (localized disease, metastatic disease, relapse, or remission). The results show the expression of β3-AR on the surface of CD45^−^ CD99^+^ PB cells, confirming the presence of β3-AR on tumour cells derived from Ewing sarcoma patients. The preliminary results also show differences in the β3-AR expression between CTCs derived from metastatic and non-metastatic patients, and suggest a possible role of β3-AR as a new marker to be combined with CD99 for the prediction of recurrence or disease severity in Ewing sarcoma patients.

## 2. Materials and Methods

### 2.1. Human Blood Samples and Biopsies

The preliminary data obtained from the clinical study included the analysis of 20 EWS patients referred to the Oncology Hematology Units of Meyer Children’s Hospital (Florence, Italy) and Azienda Ospedaliero-Universitaria Pisana (Pisa, Italy). The patients were included in the study after X-rays, magnetic resonance imaging (MRI) and positron emission tomography (PET)-TC scan evaluation, together with histological examination of a needle biopsy sample and analysis of EWS-FLI1 translocation that resulted in a diagnosis of Ewing Sarcoma. All subjects, or their legal tutors if minors, gave their informed consent for inclusion before they participated in the study. The study was conducted in accordance with the Declaration of Helsinki, and the protocol was approved by the Pediatric Ethics Committee of Regione Toscana (Project identification code “Beta 3 2018”, ethical permission code 34/2018, permission date 27 March 2018). Peripheral blood samples derived from EWS patients diagnosed before and during the clinical study (2018–2020) were collected according to clinical management. Moreover, for 14 patients bone marrow (BM) blood was also available during the study, while the biopsy samples were collected only from 7 patients. Finally, we included in the study 5 PB samples derived from cases referred to Meyer Children’s Hospital that after all the steps of the clinical evaluation received a non-oncological diagnosis, they were thus excluded from the group of patients and evaluated as healthy samples. All the blood samples were collected in EDTA coated tubes and processed within few hours, whereas biopsy samples were preserved in MACS Tissue Storage Solution (Miltenyi Biotec, Bergisch, Gladbach, Germany) and processed within few hours. Overall, we included in the study 20 EWS patients and analysed 20 PB samples, 14 BM samples, and 7 biopsies. In order to gain insight on the significance of analysed markers, biological samples were divided into 2 different group: Metastatic (M) those derived from patients affected by metastatic disease at onset and non-metastatic (NM) those derived from patients with localized disease at diagnosis. Based on this partition we analysed PB samples derived from 10 metastatic and 10 non-metastatic patients, while regarding the evaluation of the other biological samples, we analysed MB samples derived from 5 metastatic and 9 non-metastatic patients and bioptic samples derived from 4 metastatic and 3 non-metastatic patients.

### 2.2. Preparation of Blood Samples for Cytofluorimetric Analysis

**Figure 1:**
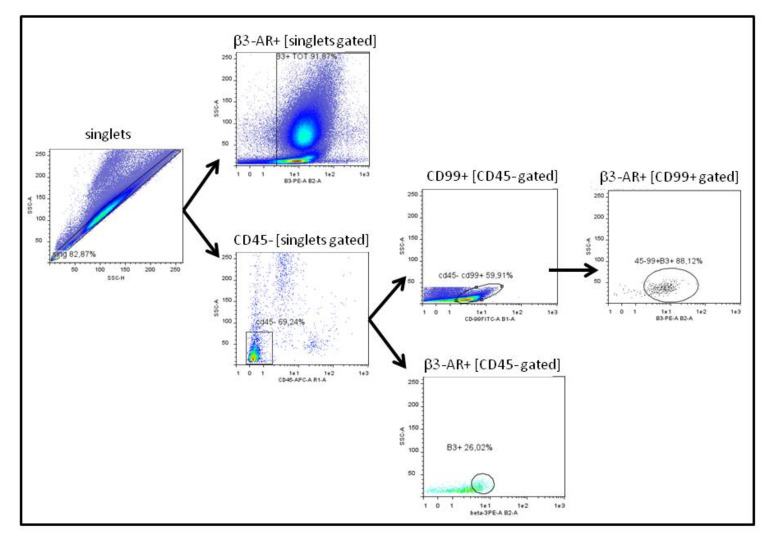
The figure represents the gating strategy used for the cytofluorimetric analysis of all samples.

Briefly, BM and PB samples were subjected to lysis of red blood cells with 10 volumes of Red Blood Cell Lysis Solution 1× (Miltenyi Biotec, Bergisch, Gladbach, Germany), incubated at room temperature for 15 min, then washed twice with MACSQuant Running Buffer (Miltenyi Biotec, Bergisch, Gladbach, Germany), and centrifuged at 1200 rpm for 5 min. Then, cellular pellet was resuspended in MACSQuant Running Buffer and labelled with the specific antibodies: β3-PE conjugated antibody (ab102778, Abcam, Cambridge, UK) and CD45-APC (Miltenyi Biotec, Bergisch, Gladbach, Germany). After 15 min of incubation at 4 °C, samples were washed and the total amount of cells was then analysed with MACSQuant FACS instrument (Miltenyi Biotec, Bergisch, Gladbach, Germany). After the first evaluation of β3-PE positive cells within all blood cells collected, an amount of maximum 10^7^ cells were depleted from CD45^+^ cells: Samples were magnetically labelled with CD45 Microbeads (Miltenyi Biotec, Bergisch, Gladbach, Germany) and split into two different populations, the CD45^+^ and the CD45^−^ fraction, by using the AutoMACS Separator (Miltenyi Biotec, Bergisch, Gladbach, Germany) following the manufacturer’s instructions. The fraction of CD45^−^ cells was centrifuged at 1200× rpm for 5 min, resuspended in MACSQuant Running Buffer, and labelled with the fluorescent antibodies β3-PE, CD45-APC, and CD99-FITC following the same steps described above. The β3-PE and CD99-FITC positive cells were then analysed with MACSQuant FACS instrument (Miltenyi Biotec, Bergisch, Gladbach, Germany). The gating strategy used for the analysis of samples is represented in Figure 1, briefly: (i) singlets were gated to eliminate aggregate cells before each analysis; (ii) on all singlets gated cells percentage of β3-AR^+^ cells was evaluated; (iii) after depletion of CD45^+^ cells, on singlets gated cells, CD45^−^ cells was gated to eliminate eventual CD45^+^ residual cells; (iv) on CD45^−^ gated cells, CD99^+^ cells were gated and evaluated; (v) on CD99^+^ gated cells % of β3-AR^+^ cells were evaluated; and (vi) % of β3-AR^+^ cells was evaluated also on all CD45^−^ gated cells.

### 2.3. Biopsies Samples

EWS biopsies, preserved in MACS Tissue Storage Solution, were dissociated in single cells with Tumour Dissociation Kit (Miltenyi Biotec, Bergisch, Gladbach, Germany) by GentleMACS instrument (Miltenyi Biotec, Bergisch, Gladbach, Germany), following the manufacturer’s instructions. At the end of the protocol, samples were labelled with fluorescent antibodies, such as β3-PE, CD45-APC, and CD99-FITC, and analysed by flow cytometry following the same steps described for blood samples and the same gating strategy shown in Figure 1.

### 2.4. In Vitro Analysis of CD99 Expression after β3-ARs Blockade

Human Ewing sarcoma cell lines A673 were cultured in DMEM high glucose medium (Euroclone Group, Pero, Italy) supplemented with 10% fetal bovine serum (FBS), 1% of l-glutamine, and 1% of penicillin-streptomycin, and SK-ES-1 cell lines were cultured in McCoy’s 5A Medium (Sigma-Aldrich, St. Louis, MO, USA) supplemented 15% FBS, 1% of l-glutamine, and 1% of penicillin-streptomycin. Cells were maintained at 37 °C in a 5% CO_2_ humidified atmosphere incubator. Cells were treated with SR59230A (Sigma-Aldrich, St. Louis, MO, USA) at 5 M and 10 M for 24 h. To detect cells viability MTT assay was performed as described previously [19]. For the analysis of CD99 expression, after 24 h of SR59230A treatment, cells were washed and labelled with CD99-FITC antibody as described above. CD99-FITC positive cells and the CD99 MFI were then analysed with MACSQuant FACS instrument and software (Miltenyi Biotec, Bergisch, Gladbach, Germany).

### 2.5. Statistical Analysis

Statistical analysis was performed using Graph Pad Prism software (GraphPad, San Diego, CA, USA) by Student’s *t*-test.

## 3. Results

The study focused on the investigation of β3-AR expression on CTCs isolated from EWS patients. In order to gain insight on the significance of analysed markers, biological samples were divided into two different group: Metastatic those derived from patients affected by metastatic disease at onset and non-metastatic those derived from patients with localized disease at diagnosis (as described in methods section). Since CD99 antigen is also expressed in synovial sarcoma and low-grade fibromyxoid sarcoma, patients were included in the study only after the clinical confirmation of Ewing sarcoma diagnosis, as described in method section. As CD99 antigen is expressed on the surface of EWS cells, it was used for detection and isolation of CTCs in EWS PB. Besides tumour cells, CD99 antigen is also expressed on the surface of leukocytes population (CD45^+^), thus for the analysis of β3-Ars expression on EWS tumour cells it was necessary to evidence the CD45^+^ population to deplete it and enrich for CD45^−^ CD99^+^ tumour cells. Moreover, for some patients, a biopsy sample and bone marrow blood was also available, thus the analysis of β3-AR expression was extended to the cells derived from the other biological samples to verify the presence of β3-ARs positive cells in the original tumour site and in microenvironment.

### 3.1. Analysis of Peripheral Blood Derived Cells

**Figure 2:**
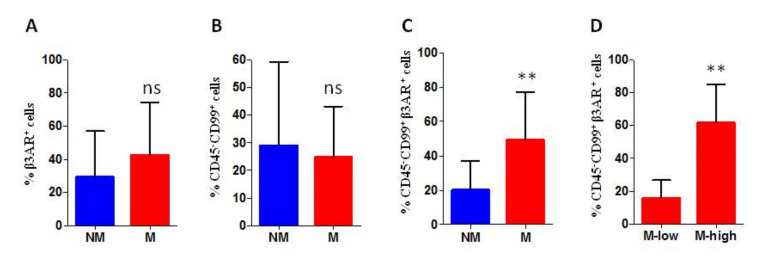
Cytofluorimetric (FACS) analysis of peripheral blood derived cells, the percentage of positive cells is reported. (**A**) percentage of beta-3-adrenergic receptor (β3-AR) positive cells within of all peripheral blood (PB) cells (% β3-AR^+^ cells range: Non-metastatic (NM) 11–58%; metastatic (M) 25–75%); (**B**) percentage of CD99 positive cells on the surface of CD45^−^ PB cells (% CD99^+^ cells range: NM 0–59%; M 8–43%); (**C**) percentage of β3-AR positive cells on CD45^−^ CD99^+^ gated cells (% β3-AR^+^ cells range: NM 0–39%; M 5–80%); and (**D**) percentage of β3-AR positive cells on CD45^−^ CD99^+^ gated cells analysed within the group of metastatic patients (% β3-AR^+^ cells range: Mhigh 37–80%; Mlow 5–23%). M low: M group with low β3-AR expression level, and M high: M group with high β3-AR expression level. PB from 20 patients: 10 M and 10 NM. ns: Not significant. *p* values for analysis ** *p* < 0.01.

Examination of β3-AR expression was initially performed on all PB cells. The first interesting datum we observed was that β3-AR was highly expressed on PB cells of all patients with a median value of 42% of β3-AR positive cells on the total of analysed cells. This value was largely above the one resulted within the group of healthy cases that is 8.5%. Among the group of patients, the levels of β3-AR expression on all PB cells did not show a statistically significant difference between PB cells derived from metastatic and non-metastatic patients. (Figure 2A).

**Figure 3:**
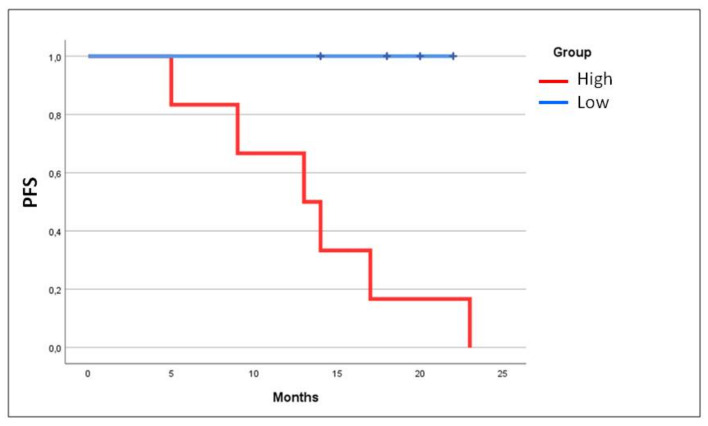
Kaplan Meier curve analysis showing the progression free survival (PFS) in metastatic patients divided in the two subgroups of high and low β3-AR expression. The curve shows the positive association between high β3-AR levels and progression of the disease. High: Metastatic patients with percentage of β3-AR positive cells higher than 30%; Low: Metastatic patients with percentage of β3-AR positive cells lower than 30%.

After depletion of CD45^+^ cells, CD45^−^ PB cells were analysed for CD99 and β3-AR expression. The analysis of CD99 marker on CD45^−^ PB cells show a high level of expression in 15 out of 20 patients without remarkable differences between metastatic and non-metastatic patients (Figure 2B), while the investigation of β3-AR expression on CD45^−^ CD99^+^ gated cells revealed a higher β3-AR expression in samples derived from metastatic patients, with a significant statistical difference (Figure 2C). Observing the percentage of CD45^−^ CD99^+^ β3-AR positive cells in all patients, we noticed that almost all non-metastatic cases (9/10) had a value of β3-AR positive cells below the 30%, while for metastatic patients the group seemed to be divided in two subgroups: Patients with a percentage of β3-AR^+^ cells higher and lower than 30%. Interestingly, the analysis of β3-AR expression within the metastatic patients, revealed a positive association between high β3-AR levels and progression of the disease: The patients that had a percentage of β3-AR^+^ cells higher than 30% had recurrence within the time of the study, while metastatic patients with a lower number of β3-AR^+^ cells were in complete remission (Figure 2D and Figure 3).

### 3.2. Analysis of Bone Marrow Derived Cells

**Figure 4:**
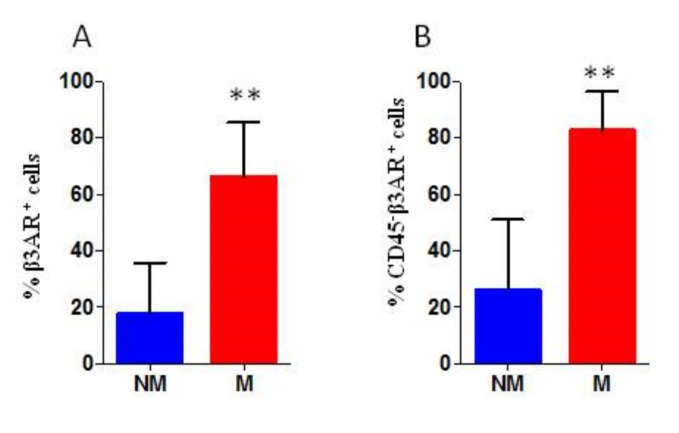
Cytofluorimetric analysis of bone marrow derived cells, the percentage of positive cells is reported. (**A**) percentage of β3-AR positive cells within all BM cells (% β3-AR^+^ cells range: NM 2.5–48%; M 46–85%) and (**B**) percentage of β3-AR positive cells within CD45^−^ gated BM cells (% β3-AR^+^ cells range: NM 0.5–56%; M 65–91%). BM from 14 patients: 5 M and 9 NM. *P* values for analysis: ** *p* < 0.01.

Since morphological analysis revealed the presence of infiltrating tumour cells in BM samples of only two patients, the analysis of β3-AR levels on CD45^−^CD99^+^ cells was not performed. Investigation of β3-AR expression on all BM derived cells revealed a higher β3-AR expression on the surface of all cells derived from metastatic patients, with a significant statistical difference (Figure 4A). Furthermore, even the evaluation of β3-AR expression on CD45^−^ gated BM cells revealed a higher β3-AR expression in samples derived from metastatic patients, with a significant statistical difference (Figure 4B). β3-AR expression on CD45^−^ gated cells could indicate an involvement of microenvironment.

### 3.3. Analysis of Biopsies Derived Cells

**Figure 5:**
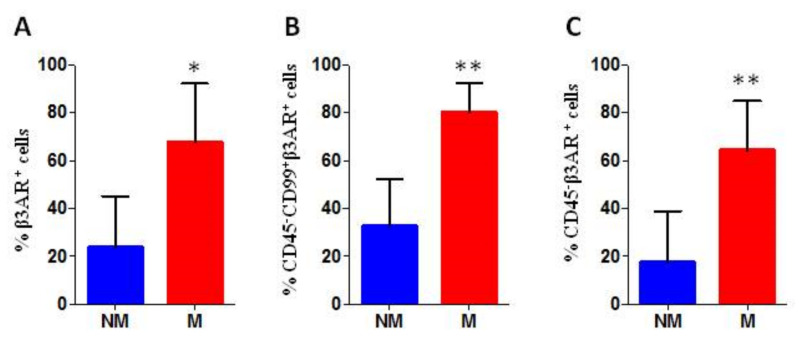
Cytofluorimetric analysis of biopsies derived cells, the percentage of positive cells is reported. (**A**) percentage of β3-AR positive cells within all cells derived from the biopsy (% β3-AR^+^ cells range: NM 6–44%; M 43–87%); (**B**) percentage of β3-AR positive cells within CD99^+^ gated cancer cells (% β3-AR^+^ cells range: NM 18–53%; M 63–86%); and (**C**) percentage of β3-AR positive cells within CD45^−^ gated cells (% β3-AR^+^ cells range: NM 4–39%; M 43–84%). Biopsy samples from 7 patients: 4 M and 3 NM. *p* values for analysis: * *p* < 0.05 and ** *p* < 0.01.

Investigation of CD45, CD99, and β3-ARs expression was performed on cells derived from the dissociated tumour biopsy. The cytofluorimetric (FACS) analysis revealed a higher total of β3-AR expression on the surface of all cells in the samples (β3-AR^+^ cells) derived from metastatic patients, with a significant statistical difference (Figure 5A). The evaluation of β3-ARs expression on tumour cells (CD45^−^ CD99^+^ gated cells) revealed a higher percentage of β3-AR positive cells in samples derived from metastatic patients, with a significant statistical difference (Figure 5B). Finally, the analysis of β3-ARs expression on tumour and microenvironmental cells (CD45^−^ gated cells) revealed a higher β3-ARs expression in samples derived from metastatic patients, with a significant statistical difference indicating a possible involvement of β3-ARs in tumour aggressiveness (Figure 5C).

### 3.4. Analysis of CD99 Expression on Ewing Cell Lines after β3-ARs Blockade

**Figure 6:**
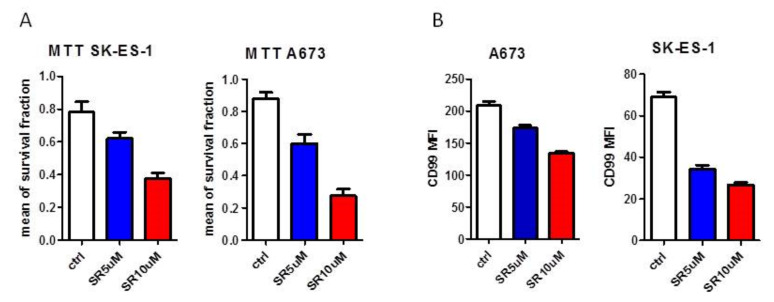
In vitro analysis of CD99 expression after β3-ARs blockade. (**A**) analysis of cellular viability with MTT survival experiment in A673 and SK-ES-1 cell lines and (**B**) CD99 mean fluorescence intensity (MFI) in A673 and SK-ES-1 cells investigated by cytofluorimetric analysis, after 24 h-treatment with SR59230A 5 M (SR5uM) and 10 M (SR10uM); ctrl: Untreated.

In order to gain insight into the relationship between CD99 and β3-ARs pathways we evaluated the expression of CD99 antigen after the treatment with the β3-ARs antagonist SR59230A in two different Ewing cell lines (A673, SK-ES-1). As expected, since β3-ARs blockade had been reported to affect tumour cell proliferation, SR59230A treatment reduced the tumour cells viability (Figure 6A) in both cell lines. Interestingly, the CD99 mean fluorescence intensity (MFI) decreased after SR59230A treatment with respect to control untreated cells (Figure 6B), showing a reduction of CD99 antigens expressed on cell surface, thus suggesting a role of β3-AR in regulating the expression of CD99.

## 4. Discussion

Ewing sarcoma is a rare tumour that affects bones and soft tissues with a high aggressive systemic disease displaying varied outcomes due to the elevated incidence of relapse and metastasis extending from isolated pulmonary metastasis to multi organ diseases widely disseminated. Usually, patients have heterogeneous diseases with outcomes that varied between 10–40%. To detect recurrence or metastasis, current methods depend largely on clinical exams, radiographic imaging, and positron emission tomography that identify areas of tumour growth or increased metabolic activity and that gives evidences of disease when the relapse is already present or advanced. Bone scan and PET help to determine if the tumour has widespread in other regions of the body, but the biopsy remains essential to be truly certain of its presence. In this work we propose β3-AR to be combined with CD99 as a possible predictor marker of recurrence or disease severity not only in the biopsy but also in circulating tumour cells. The presence of CTCs can provide valuable information about tumour composition, invasiveness, drug susceptibility, and therapeutic strategy by modulation of different drug administration [37]. As suggested by several evidences, cancer spread and tumour relapse can be predicted by the presence of CTCs. This is one of the reasons why the analysis of CTC is considered a minimally invasive method for marker detection [38]. CTCs have been identified in PB of patients affected by different types of tumours, these cells resulted to have significant clinical utility, most commonly, in epithelial malignancies (colon carcinoma, breast cancer, and prostate cancer) [16]. In these types of tumour, the presence and the number of CTCs were correlated with the recurrence and the stage of the disease. However, the utility of CTCs analysis in paediatric cancer is still controversial. Ewing sarcoma cells have a high and specific expression of the CD99 antigen. The evaluation of its expression, associated with that of the fusion protein (i.e., EWS-FLI1), allows us, today, to have a certain diagnosis for this type of neoplasm, in this study we could not perform the molecular confirmation of the fusion gene. Many studies performed the investigation of the fusion proteins EWSR1/FLI1 and EWSR1/ERG as disease markers through reverse transcription-polymerase chain reaction. Particularly, ctDNA is a valuable addition for assessment of therapy response in patients with unfavourable distributed disease. EWS-FLI1 fusion gene is the most thoroughly studied since it is the most common, it may contribute to the origin of EWS pathologies [39], thus EWS-FLI1 protein is considered a potential therapeutic target. Other genes have been described as involved in the translocation and EWS fusion: *ERG* in 5–10% of cases, and *FEV*, *ETV1,* and *ETV4* in less than 1% of cases [40]. Therefore, the identification of the translocation in CD99^+^ cells is a sure method to identify EWS cells, but there still remains the possibility of false ES negative results. A potential method to detect CD99^+^ cells within the CD45^−^ cell population is the use of flow cytometry. To identify and capture the CTCs, we need extremely sensitive and specific methods, which usually consist of a combination of enrichment and detection procedures. CD99 is also expressed on leukocytes and other normal human tissues such as the testis, haematopoietic tissues, gastric mucosa, and prostate [12,13]

Despite CTCs having been found in PB of EWS patients, their presence has not been associated with metastasis and outcome, since, until now, there are not evidences that tumour cells detected in PB patients have the ability to give rise to metastasis [16]. On the other hand, some studies reported the detection of CTCs mostly in bone marrow samples of patients identified with metastatic disease and have been associated with poor prognosis [18]. Thus, the true biological meaning or clinical relevance of detecting CTCs in EWS patient’s blood remains unknown.

**Figure 7:**
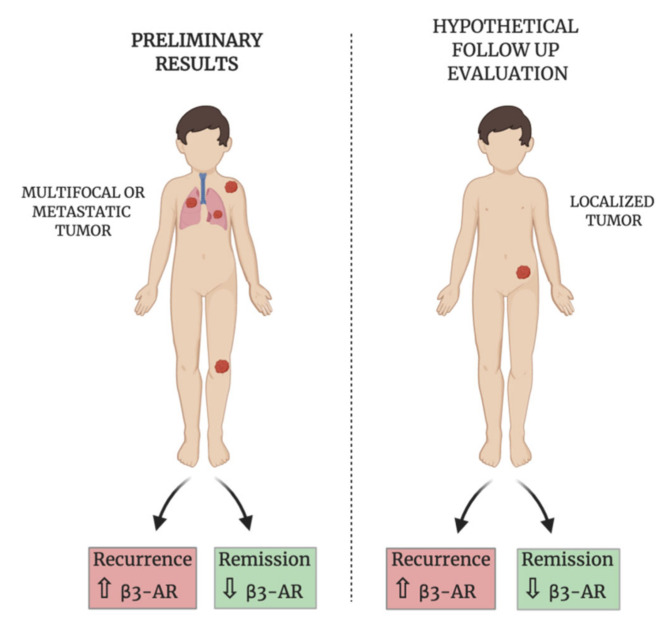
Schematic representation of the preliminary results on metastatic patients (left) and the suggested evaluation during the follow-up even for localized patients (right).

This work showed the presence of high levels of CD45^−^CD99^+^ in EWS patients, confirming the presence of CTCs in this type of paediatric cancer. We have to specify that besides EWS CTCs, the CD45^−^CD99^+^ cell population can comprehend variable levels of osteoblasts and mesenchymal stem cells, on the other hand we observed that among the patients analysed there are some cases without any expression of CD45^−^CD99^+^ cells, suggesting that even the percentage of circulating osteoblasts and/or mesenchymal stem cells is very low. Thus, we suppose that a high level of CD45^−^CD99^+^ can correspond to a well-detectable amount of CTCs differently from other solid tumours. The analysis of CD45^−^CD99^+^ expression on all PB cells did not show a statistically significant difference between metastatic and non-metastatic patients even if at diagnosis, the metastatic status is the strongest prognostic factor [3]. It is reported that patients with metastatic disease show poor outcomes and when metastasis spread to bone and bone marrow, the prognosis will be even worse [41]. Unfortunately, patients with metastatic disease often present resistance to chemotherapeutic agents [42], but the clinical data demonstrated that even localized tumour can relapse. Interestingly, the investigation of β3-ARs expression on CD45^−^CD99^+^ cells showed that the presence of a high or low β3-ARs level could be useful to further separate into subgroups. We observed that metastatic patients with low β3-ARs expression in circulating CD99^+^ cells went into complete remission during the two year of the study, while metastatic patients with high β3-ARs expressing CD99^+^ cells had relapse within the study. This could suggest a role of β3-ARs in the disease progression of Ewing sarcoma and based on these initial data, we hypothesize β3-ARs expression to be investigated as a possible prognostic marker for disease relapse. We have to highlight that our results are absolutely preliminary, and the clinical study needs a significant increase in the sample size to reach a statistical strength. As regarding the group of localized patients, the same association was not observed because of the absence of relapsing patient in the two years of the study, even if one patient had a high percentage of CD45^−^CD99^+^β3-AR^+^ cells. We cannot exclude the existence of the same positive association even in non-metastatic patients but with a different timing of relapse. Thus, it can be suggested to investigate CD45^−^CD99^+^β3-AR^+^ expression during the follow-up of all patients. (Figure 7).

Moreover, we evidenced that β3-ARs expression on all bone marrow derived cells was higher in metastatic patient and the same result was observed on CD45 negative cells. Furthermore, even the analysis of β3-ARs expression on cells derived from the biopsy samples (CD45^−^CD99^+^ and CD45^−^ cells) revealed a higher β3-ARs expression on cells obtained from metastatic patients. CD45^−^ cells population in BM and biopsy samples includes all tumour and non-tumour cells of microenvironment. Together these data suggest an intriguing role of β3-ARs expressed by bone marrow stromal cells and tumour microenvironment in the disease severity. Finally, since β3-ARs is highly expressed in adipose tissue and, as it is well known, tumour microenvironment has a fundamental role in cancer progression, we would speculate that adipose tissue in the tumour microenvironment with high β3-ARs expression could promote progression of EWS tumour [24,43].

## 5. Conclusions

**Figure 8:**
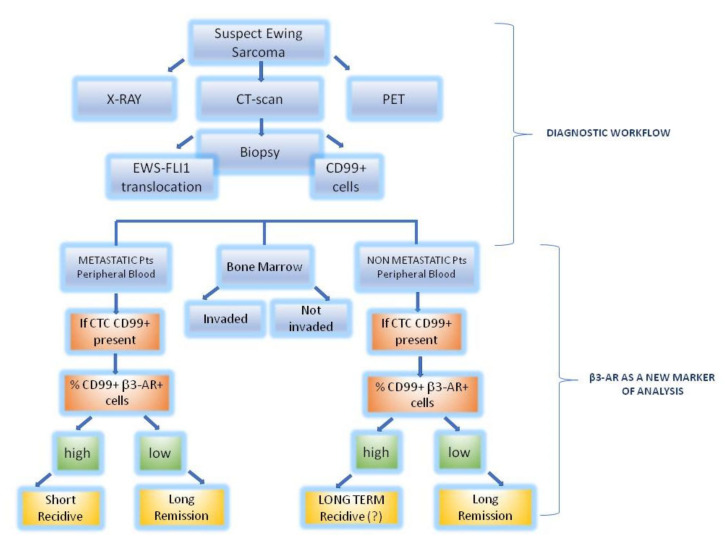
The figure represents in blue squares the routinely workflow for diagnosis and in the coloured squares the new prognostic workflow suggested according to our preliminary results.

Here we report the absolutely preliminary results of a clinical study in which we evaluated the expression of β3-ARs on EWS tumour and microenvironmental cells and hypothesize that the investigation of its levels on CD99^+^ cells could be used as a marker of disease recurrence during the follow-up of patients independently from the number of circulating CD99^+^ cells. Moreover, we identified a higher β3-ARs expression even on the surface of microenvironmental cells, suggesting that even in microenvironment the investigation of β3-ARs expression could be useful as a marker of “stromal malignancy”, intended as an indicator of the active role that stromal cells have in disease progression. For the further development of the clinical study we are expanding the enrolment of patients in order to overcome the limitation of a small sample size that has affected this preliminary study, and we are setting up the molecular analysis to confirm the presence of EWS-ETS translocation. Once the preliminary data can be confirmed by a more exhaustive study on a larger sample size, it would be possible to build a prognostic flow diagram that provides for the measurement of the β3-ARs expression in blood samples (Figure 8). For these reasons further studies need to be processed in the future. The lack of improvement in outcomes using current strategies suggests that alternative approaches are essential to make any further progress.
